# Sensory and Metabolomic Analysis Reveal the Quality Evolution of Liupao Tea During Long-Term Aging

**DOI:** 10.3390/foods15111851

**Published:** 2026-05-23

**Authors:** Haitian Ye, Xiaohui Jiang, Jinchi Tang, Jianlong Li

**Affiliations:** 1 College of Coastal Agricultural Sciences, Guangdong Ocean University, No. 1 Haida Road, Mazhang District, Zhanjiang 524088, China; haitianye@126.com; 2 Tea Research Institute, Guangdong Academy of Agricultural Sciences, No. 6 Dafeng Road, Tianhe District, Guangzhou 510640, China; 3 Guangdong Provincial Key Laboratory of Tea Plant Resources Innovation and Utilization, No. 6 Dafeng Road, Tianhe District, Guangzhou 510640, China; 4 College of Food and Pharmaceutical Engineering, Wuzhou University, No. 82 Fumin 3rd Road, Wuzhou 543002, China; jiangxiaohui_tea@163.com; 5 Guangxi Liupao Tea Modern Industry College, Wuzhou University, No. 82 Fumin 3rd Road, Wuzhou 543002, China

**Keywords:** Liupao tea, non-volatile metabolites, tea liquor color, taste, correlation analysis

## Abstract

Liupao tea develops characteristic sensory properties during aging. However, the evolution of non-volatile metabolites and their relationship with sensory quality remain unclear. Here, three samples aged for 8, 13, and 20 years were analyzed using electronic tongue, colorimetry, and metabolomics. Tea liquor brightness (L*) increased with aging, whereas redness (a*) and yellowness (b*) decreased. Strong correlations between taste and color were observed. Catechins and free amino acids peaked at the intermediate stage, while alkaloids varied, with caffeine remaining stable. A total of 1897 metabolites were identified, with flavonoids increasing and terpenoids and lipids decreasing during aging. The intermediate stage represented a critical transition point with metabolic reprogramming. Key sensory-related metabolites included theobromine, glutamic acid, and theanine (associated with umami, sweetness, and color), as well as gallocatechin gallate, catechin gallate, theacrine, aspartic acid, and arginine (linked to bitterness and yellowness). Flavonoid- and terpenoid-rich modules were also identified. All samples were from a single producer and may not represent all Liupao teas. Overall, this study reveals coordinated changes in sensory quality and metabolites during Liupao tea aging.

## 1. Introduction

Liupao tea, a typical Chinese dark tea, is well known for its unique sensory characteristics, described as “red, thick, aged, and mellow”, and it exhibits various health benefits, including lipid-lowering and antioxidant activities [1,2]. The quality formation of Liupao tea is a complex biochemical process influenced by multiple factors, including tea cultivar, processing technology, microbial communities, and aging time [3,4,5]. Among these, aging is an indispensable stage that plays a crucial role in flavor maturation and quality improvement. During aging, a series of transformations occur in tea components through the action of microorganisms and enzymes. These changes lead to significant alterations in appearance, liquor color, aroma, and taste, ultimately forming the characteristic aged aroma and mellow flavor of Liupao tea [6,7,8]. At present, studies on the effects of aging time on Liupao tea quality have made some progress. Comparison among samples stored for 0, 3, 5, 8 and 10 years showed that, within a certain aging period, prolonged aging darkens the appearance, enhances red and bright liquor color, mellows taste from bitter/astringent to smooth, and shifts aroma from dull to woody and aged [7]. In terms of chemical composition, long-term aging (about 8~10 years) reduces free amino acids and tea polyphenols, promotes theabrownin (TB) accumulation, and alters pathways such as catechin oxidation, flavonoid glycosylation/methylation, and alkaloid formation [6,7,8]. These transformations of quality-related metabolites collectively shape the unique flavor characteristics of Liupao tea. However, the systematic variation patterns of non-volatile metabolites under different aging times, especially for longer aging periods (>15 years), remain insufficiently understood. In addition, key metabolites linked to sensory changes remain unclear [7,9], limiting precise determination of the optimal aging period of Liupao tea.

In recent years, with advances in analytical techniques, molecular sensory science and metabolomics have been widely applied in tea quality research. For example, the electronic tongue simulates human taste perception through sensor arrays, reducing subjective bias and enabling accurate identification of taste attributes [10,11]. A colorimeter quantitatively characterizes tea liquor color by measuring L*, a*, and b* values [12]. In the field of Liupao tea research, electronic tongue has been used to objectively quantify taste attributes, while colorimetry has been applied to precisely evaluate liquor color changes [12,13,14]. In addition, many studies combine targeted and untargeted metabolomics to systematically identify and quantify dynamic changes in metabolite profiles during tea processing and aging [7,14,15,16]. These approaches clarify the chemical basis of Liupao tea quality. Here, we integrate electronic tongue, colorimetry, and targeted and untargeted metabolomics to systematically assess the effects of extended aging on non-volatile metabolites. By correlating chemical data with sensory attributes, this study aims to identify key candidate metabolites closely associated with long-term aging quality. These findings will clarify whether longer aging improves quality and provide a basis for quality control, storage, and optimal drinking time of Liupao tea.

## 2. Materials and Methods

### 2.1. Materials and Chemicals

Three Liupao tea samples from Wuzhou Zhongming Jinliubao Tea Co., Ltd (Wuzhou, China) were investigated. With aging initiation years of 2005, 2012, and 2017, corresponding to 20, 13, and 8 years of aging, respectively, these samples were designated as ZM2005, ZM2012, and ZM2017. The samples were selected on practical grounds, and their storage intervals were irregular. In addition, all samples were processed using the cold-water pile fermentation method and aged under the humid and constant-temperature conditions of an air-raid shelter. Eight catechin monomer standards were purchased from Zhenzhun Biotechnology Co., Ltd. (Shanghai, China). Four alkaloid standards, including caffeine (≥98%), theobromine (≥98%), theacrine (≥99%), and theophylline (≥98%), were obtained from Shanghai Yuanye Biotechnology Co., Ltd. (Shanghai, China). Four theaflavin (TF) monomer standards were purchased from Shanghai Zzbio Co., Ltd. (Shanghai, China). Thirty amino acid standards were purchased from Guangzhou Baohui Biotechnology Co., Ltd. (Guangzhou, China).

### 2.2. Electronic Tongue Measurements

The taste profiles of the samples were characterized using an α-ASTREE electronic tongue system (Alpha MOS, Toulouse, France), equipped with an Ag/AgCl reference electrode and a set of seven selective sensors (SRS, SWS, BRS, STS, UMS, GPS, and SPS). The electronic tongue system employed measures electrochemical potentials generated by cross-reactive sensor arrays. The sensor array responds to different taste-related chemical compounds and collectively evaluates taste attributes, including sourness, sweetness, bitterness, saltiness, umami, and other complex flavor perceptions. The measurement protocol was established by refining previously published procedures, with necessary adjustments introduced to optimize performance under the present experimental conditions [17]. Liupao tea powders (2 g) were taken in a beaker, extracted in boiling water (100 mL) for 5 min. The tea infusion was quickly filtered through gauze, followed by filtration using filter paper, and set aside for analysis. Prior to measurement, the sensors were activated and calibrated with standard solutions. A 25 mL aliquot of the prepared sample was placed in a specialized beaker for electronic tongue analysis. The acquisition time was set at 120 s with a frequency of 1.0 Hz and a stirring rate of 1. The measurement adhered to the principle of “measure once, clean once”. A computer recorded the response of the electronic tongue every second. Each tea sample infusion was repeatedly measured 7 times, and the 3 stable data points were selected for analysis.

### 2.3. Chromatic Difference Analysis

The color properties of Liupao tea infusions were analyzed using a YS6010 desk-top spectrophotometer (Shenzhen Sanenshi Technology Co., Ltd., Shenzhen, China) with reference to a modified method [18]. Tea samples (3 g) were extracted in 100 mL of boiling water for 1, 3, and 5 min. The instrument was calibrated using standard white and black plates, with distilled water as a reference. Three biological replicates were performed in the experiment. Color values were recorded in the CIELAB system, where L* represents lightness, while a* and b* correspond to green–red and blue–yellow coordinates, respectively.

### 2.4. Analysis of Thearubigins (TRs) and TBs

The contents of TRs and TBs were determined by a spectrophotometric method based on the previous study with some modifications [18]. Tea powder (0.3 g) was extracted with 20 mL boiling water for 20 min, centrifuged, and diluted to 50 mL. Three colorimetric solutions (A–C) were then prepared using oxalic acid reaction, n-butanol partition, and ethyl acetate extraction, respectively, followed by ethanol dilution to 25 mL. Oxalic acid stabilizes the spectral characteristics of theaflavins by adjusting the acidity, while n-butanol selectively extracts theaflavins to achieve separation of the components. Together, they provide the basis for the accurate determination of tea pigment content. Absorbance was measured at 380 nm using a UV–Vis spectrophotometer (Shanghai Youke Instrumentation Co., Ltd., Shanghai, China), and TRs and TBs contents were calculated as follows: X_TR_ = (16.944 ×(A_a_ − 0.5A_c_ − A_b_))/(100 × 0.3), and X_TB_ = (16.944 × A_b_)/(100 × 0.3). Three biological replicates were performed in the experiment.

### 2.5. Analysis of Catechins, Alkaloids, and TFs

Liupao tea powders (0.2 g) were extracted with 70% methanol (10 mL, preheated to 70 °C) for 15 min at 70 °C, and then centrifuged at 3500 r/min for 10 min. The supernatant was collected, filtered through a 0.22 µm membrane, and injected into the system. Catechins, alkaloids, and TFs were determined using a high-performance liquid chromatography analyzer (Alliance, Waters, Milford, MA, USA) fitted with a ZORBAX Eclipse C18 column (4.6 mm × 150 mm, 5 μm) based on a modified method [18]. The analysis was conducted at 35 °C with an injection volume of 10 μL. A binary mobile phase comprising aqueous acetonitrile (7%) with 2% acetic acid (A) and acetonitrile with 2% acetic acid (B) was applied under gradient elution (1 mL/min). Three biological replicates were performed in the experiment. UV detection was set at 278 nm using a diode array detector, and compound identification and quantification were achieved using authentic standards.

### 2.6. Analysis of Free Amino Acids

According to the national standard method Determination of Free Amino Acids in Plants (GB/T 30987—2020) with appropriate modifications [19], the free amino acids in the samples were extracted and determined. Liupao tea powders (0.2 g) were extracted in 2.5% sulfosalicylic acid (3 mL). The sample was vortexed, sonicated for 15 min, equilibrated for 1 h, and centrifuged at 10,000 r/min for 10 min. The supernatant was filtered (0.22 μm) prior to analysis, and standard solutions were diluted tenfold before injection. Free amino acids were analyzed using an amino acid analyzer, with theanine pretreated using 2.5% sulfosalicylic acid. Separation was performed on a sodium cation-exchange column with a Sykam S433D Physiological Li C4 system (Sykam GmbH, Gewerbering, Germany), using lithium citrate buffers (pH 2.9, 4.2, and 8.0) as mobile phases. Detection wavelengths were set at 570 and 440 nm after ninhydrin derivatization. The flow rates for the mobile phase and reagent were 0.45 and 0.25 mL/min, respectively, while the column, reaction unit, and autosampler temperatures were maintained at 38, 130, and 5 °C. The injection volume was 50 μL [18]. Three biological replicates were performed in the experiment.

### 2.7. Untargeted Metabolomic Analysis

The Liupao tea samples were placed in a freeze-dryer and subjected to vacuum freeze-drying for 63 h. The dried samples were then ground into a fine powder using a grinding mill at 30 Hz for 1.5 min. Liupao tea powders (30 mg) were extracted with 70% aqueous methanol (1.5 mL) pre-cooled to −20 °C. The mixture was vortexed for 30 s every 30 min (6 times in total). After centrifugation at 12,000 r/min for 3 min, the supernatant was collected, filtered through a 0.22 μm membrane, and subjected to analysis [20]. QC samples were prepared by pooling multiple samples and analyzed every ten injections to monitor system stability. Ultra-performance liquid chromatography-tandem mass spectrometry analysis was performed with a flow rate of 0.35 mL/min, column temperature of 40 °C, and injection volume of 2 μL. The mobile phase consisted of 0.1% formic acid in water (A) and acetonitrile (B), with a gradient from 5% to 95% B over 9 min, followed by re-equilibration. Identification was conducted using an ESI source in both ion modes under optimized conditions, and data were acquired in MRM mode with nitrogen as the collision gas. Individual transitions were optimized and monitored according to retention behavior. Three biological replicates were performed in the experiment.

### 2.8. Data Analysis

Correlation analysis was conducted using an online visualization platform (https://hiplot.com.cn/cloud-tool/drawing-tool/list) following previously reported procedures (accessed on 31 March 2026) [18]. Network construction based on weighted gene co-expression analysis (WGCNA), as well as orthogonal partial least squares discriminant analysis (OPLS-DA) and corresponding permutation tests, were implemented on the Metware Cloud platform (https://cloud.metware.cn) (accessed on 15 April 2026) [21]. Heatmap visualization was generated using TBtools (Version 2.435, Guangzhou, China) [22], whereas other graphical outputs were produced via the Metware Cloud platform. Statistical analyses were performed using SPSS software (Version 24.0, IBM Corp., Armonk, NY, USA). For comparisons among multiple groups, one-way analysis of variance (ANOVA) followed by Duncan’s multiple range test was applied. Statistical significance was defined at *p* < 0.05, with *p* < 0.01 indicating a higher level of significance. All data are presented as mean ± standard deviation (SD), based on three independent biological replicates.

## 3. Results

### 3.1. Effects of Different Aging Durations on the Taste and Color Characteristics of Liupao Tea Infusion

**Figure 1:**
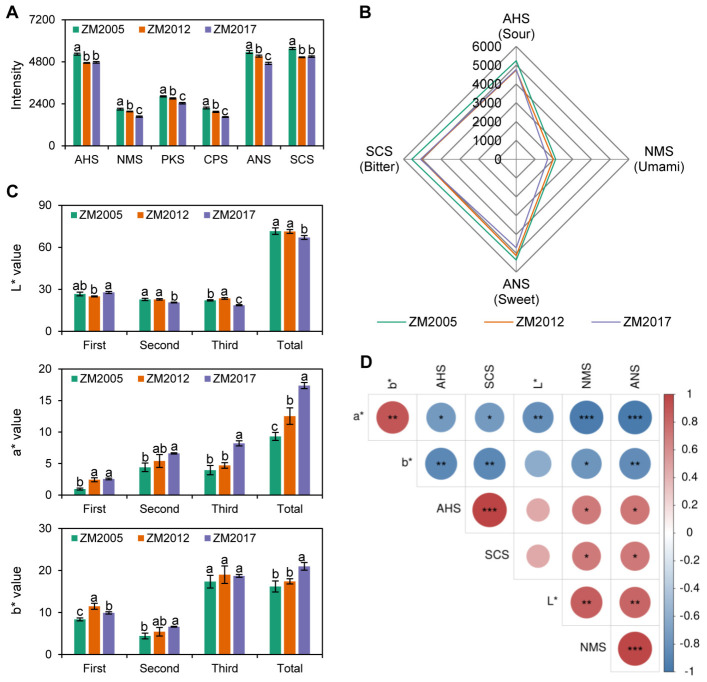
Analysis of taste and color changes in Liupao tea infusions with different aging durations. (**A**) Differential analysis of electronic tongue sensor responses. AHS represents sourness; PKS and CPS represent composite taste; NMS represents umami; ANS represents sweetness; SCS represents bitterness; L* represents lightness; a* represents the degree of greenness (negative)- redness (positive); b* represents the degree of blueness (negative)-yellowness (positive). (**B**) Radar plot analysis of major taste attributes based on electronic tongue responses. (**C**) Changes in tea liquor color parameters with aging time. (**D**) Correlation heatmap between electronic tongue responses and color parameters. The larger the dot, the higher the correlation coefficient. * indicates *p* < 0.05, ** indicates *p* < 0.01, and *** indicates *p* < 0.001. (**A**,**C**) Different letters indicate significant differences among samples (*p* < 0.05).

To objectively evaluate the effects of different aging durations on the taste and color of Liupao tea infusion, this study employed a combined analysis using an electronic tongue and a colorimeter. OPLS-DA of electronic tongue and colorimetric data showed strong model performance (R^2^X = 0.874, R^2^Y = 0.991, Q^2^ = 0.949) without overfitting, as confirmed by permutation tests (Figure S1A,B in the Supplementary Materials). In terms of taste, electronic tongue analysis showed significant differences in sensor responses among samples (*p* < 0.05), with good repeatability within samples (Figure 1A,B). The sensor array is based on field-effect transistor sensor technology. Different taste-active compounds interact with the sensor membranes through selective ionic interactions, generating distinct potential difference signals. Lower response values of the sourness (AHS), umami (NMS), and bitterness (SCS) sensors indicate higher taste intensity, whereas the sweetness (ANS) sensor shows the opposite trend. ZM2005 showed higher AHS, NMS, ANS, and SCS response values than ZM2012 and ZM2017, indicating the highest sweetness but lowest sourness, umami, and bitterness (Figure 1B). ZM2012 and ZM2017 had similar AHS and SCS responses, while ZM2017 showed lower NMS and ANS values, suggesting stronger umami but lower sweetness. For color analysis, to eliminate the influence of infusion concentration, gradient brewing times (1, 3, and 5 min) were applied for color measurement. The results showed that with longer aging, L* generally increased while a* and b* decreased, indicating that the tea liquor gradually shifted from bright orange-red to a lighter olive or gray-brown tone (Figure 1C). Differences in color parameters under varying brewing times also reflected the dynamic extraction process of tea infusion. Correlation analysis further revealed the intrinsic relationships between taste and color attributes. The L* value was positively correlated with NMS and ANS, whereas a* and b* values were negatively correlated with most taste responses (Figure 1D). These findings suggest a coordinated evolution between sensory taste and visual color during the aging of Liupao tea.

### 3.2. Effects of Different Aging Durations on Targeted Metabolites in Liupao Tea

Catechins and their derivatives, alkaloids, and amino acids are important non-volatile metabolites that influence tea quality [23]. This study systematically investigated the variation patterns of these three classes of key targeted metabolites during the aging of Liupao tea (Figure S2 in the Supplementary Materials and Table 1). The results showed that different metabolite groups exhibited coordinated but distinct patterns of change. For catechins and their derivatives, including simple catechins, esterified catechins, oxidation products such as TFs, and gallic acid, most catechin monomers and total catechin content peaked in the intermediate-aged sample (ZM2012), while lower levels were observed in both the longest-aged (ZM2005) and shortest-aged (ZM2017) samples. This non-linear “increase–decrease” pattern indicates that the intermediate stage is a critical period for catechin transformation and degradation (Table 1). Among alkaloids (caffeine, theobromine, theophylline, and theacrine), caffeine remained the most stable, theobromine peaked in the youngest sample (ZM2017), and theophylline was elevated in both ZM2005 and ZM2017, reflecting compound-specific responses to aging. For the more than 20 individual free amino acids and their total content, peak accumulation occurred in the ZM2012 sample, followed by a decline. This pattern mirrors that observed for catechins and suggests that free amino acids undergo dynamic formation and subsequent degradation during aging. In summary, catechins and free amino acids exhibited consistent peak levels at the intermediate aging stage (ZM2012), whereas alkaloids showed compound-dependent variations. These findings collectively reveal the complex chemical transformation patterns of Liupao tea during long-term aging.

### 3.3. Effects of Different Aging Durations on Untargeted Metabolites in Liupao Tea

**Figure 2:**
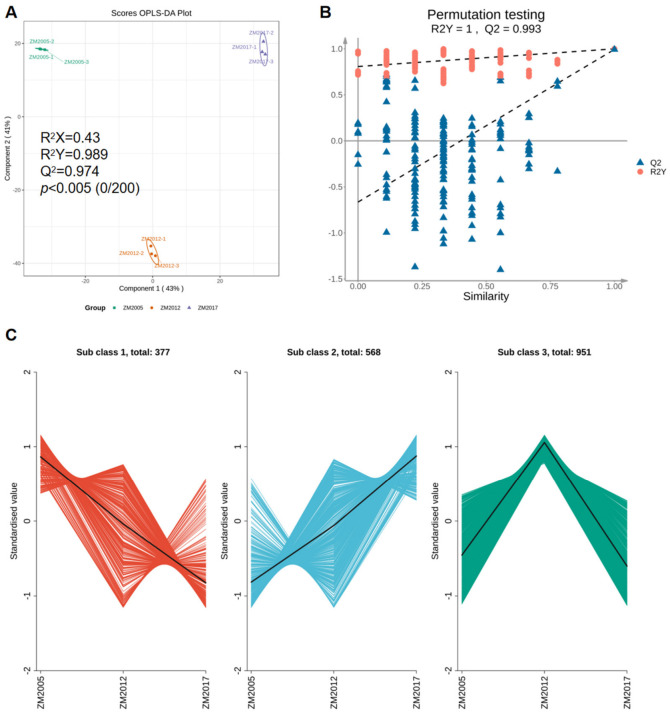
Multivariate statistical analysis of untargeted metabolomics in liupao tea with different aging durations. (**A**) OPLS-DA score plot based on widely targeted metabolomics. (**B**) Permutation test of the OPLS-DA model. (**C**) K-means clustering analysis of metabolites.

**Figure 3:**
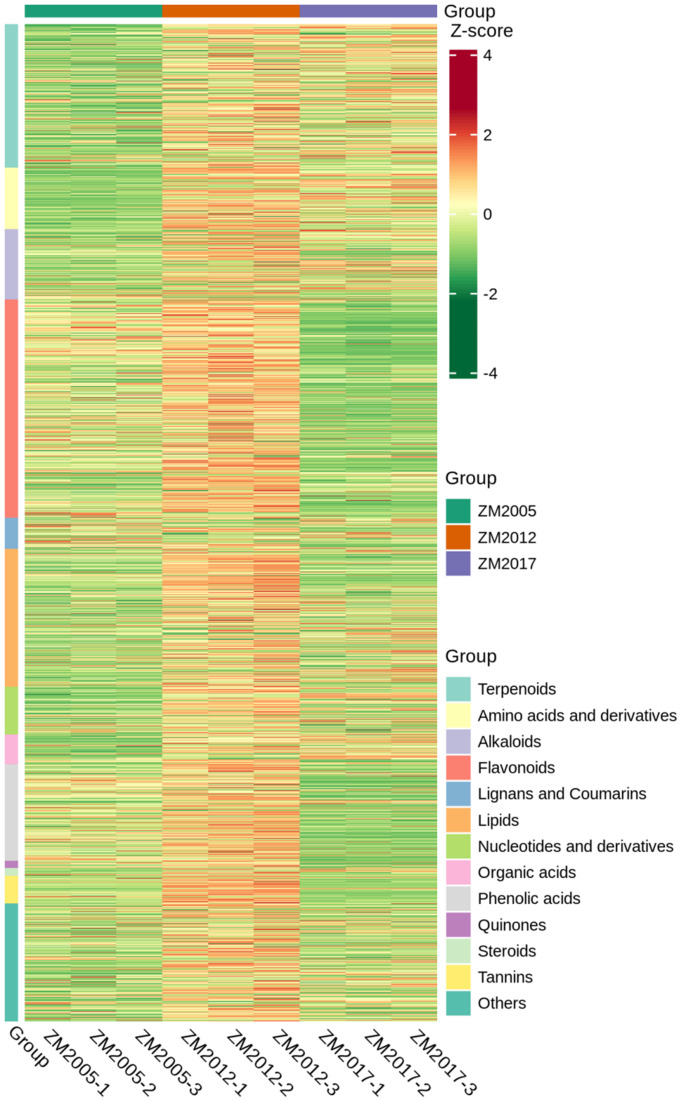
Heatmap of differential metabolites.

In this study, untargeted metabolomics combined with multivariate statistical analysis was applied to systematically investigate metabolite variations in Liupao tea with different aging durations. Data stability was first assessed using the coefficient of variation (CV). Most metabolites showed low CV values, indicating high reproducibility and reliable metabolomic data (Figure S3 in the Supplementary Materials). To distinguish metabolic differences among aging groups, OPLS-DA was performed. This supervised method effectively filters variation unrelated to classification, allowing the identification of significantly altered metabolites [16,20]. The OPLS-DA score plot clearly separated ZM2005, ZM2012, and ZM2017, demonstrating distinct metabolite compositions across aging durations (Figure 2A). A 200-times permutation test confirmed the model’s robustness and lack of overfitting (Figure 2B). K-means clustering further classified differential metabolites into subgroups with distinct variation trends, including coordinated upregulation, downregulation, and stage-specific changes during aging (Figure 2C). Heatmap analysis (Z-score) visualized the abundance patterns of key differential metabolites across samples, confirming the dynamic evolution of metabolites during aging (Figure 3). Consistent with the targeted metabolite analysis, ZM2012 showed the largest number of differential metabolites, indicating that the intermediate aging stage is a critical period of metabolic reprogramming. Overall, these results demonstrate that Liupao tea samples with different aging durations can be clearly distinguished at the untargeted metabolite level, revealing systematic and stage-specific changes in their metabolic profiles.

### 3.4. Screening of Differential Metabolites in Liupao Tea with Different Aging Durations

**Figure 4:**
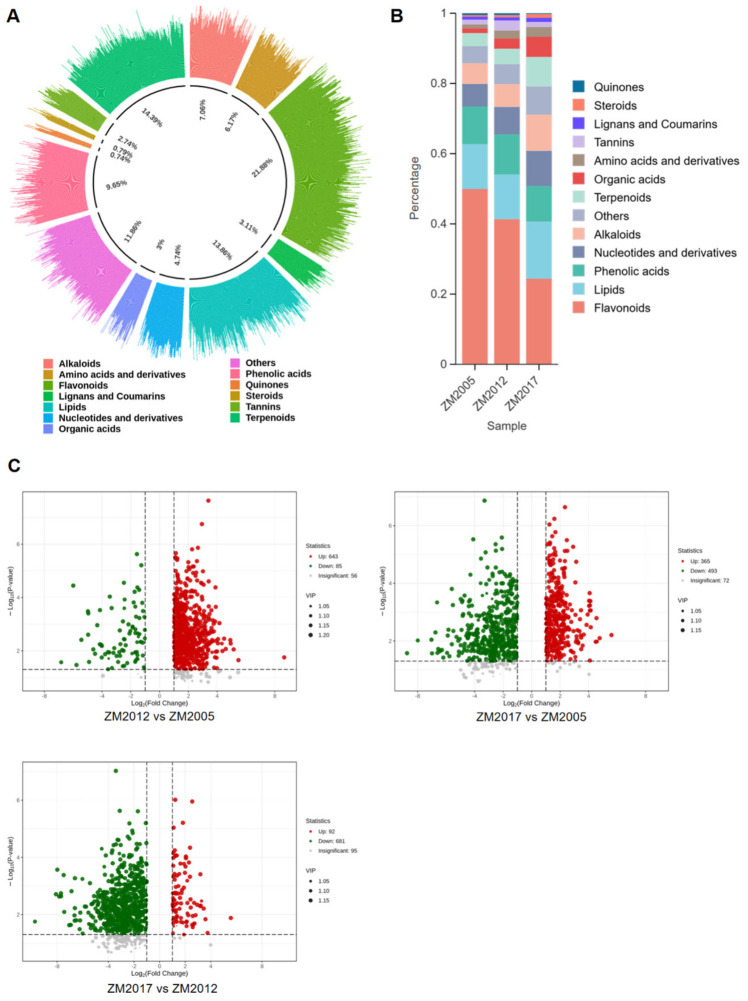
Screening of differential metabolites in liupao tea with different aging durations. (**A**) Circular classification chart of metabolites. (**B**) Bar plot of relative abundance of major compound classes. (**C**) Volcano plots of differential metabolites.

**Figure 5:**
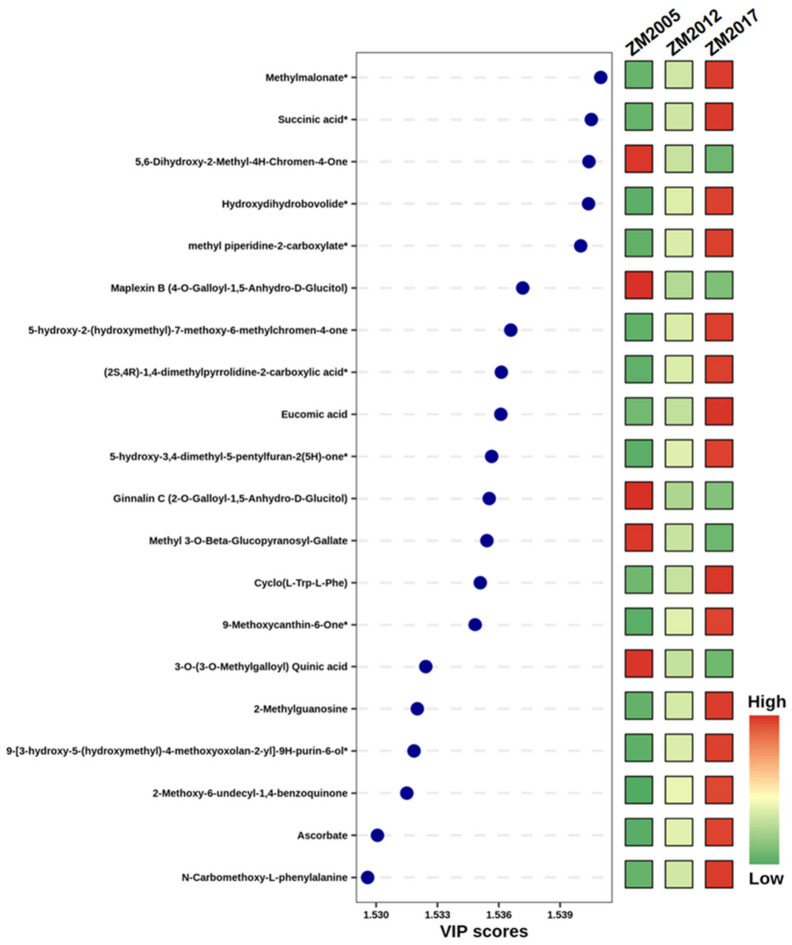
Heatmap of variable importance in projection (VIP) scores and expression patterns of key differential metabolites. * indicates that the identified metabolite has isomeric forms.

In this study, untargeted metabolomics was applied to Liupao tea samples with different aging durations to systematically screen and compare differential metabolites (Figure 4). A total of 1897 metabolites were identified, with flavonoids representing the largest proportion (21.88%), followed by terpenoids (14.39%) and lipids (13.86%) (Figure 4A). Analysis of relative abundance showed that flavonoids increased with aging, from 24.4% in ZM2017 to 49.9% in ZM2005, while terpenoids, lipids, alkaloids, nucleotides, and organic acids decreased. An increase in the proportion of flavonoids was observed in ZM2005, which is attributed to the loss of other components. Tannins initially rose and then declined (Figure 4B). Volcano plot analysis revealed significant metabolic differences among groups. Compared with ZM2005, ZM2012 and ZM2017 showed 643/85 and 365/493 upregulated/downregulated metabolites, respectively. Compared with ZM2012, ZM2017 exhibited 92 upregulated and 681 downregulated metabolites. The largest variation occurred at ZM2012, indicating a dynamic metabolic transition (Figure 4C). ZM2012 represents a key intermediate stage with the highest metabolite diversity, likely due to active microbial transformation and chemical oxidation. In contrast, ZM2017 is under-aged, while ZM2005 is over-aged with a simpler metabolic profile. However, the mechanisms underlying these changes still need further study. Using variable importance in projection (VIP) values, the top 20 key differential metabolites were selected. Heatmap patterns showed that 15 metabolites peaked in ZM2017 and decreased with aging, whereas five metabolites, including 5-hydroxy-3,4-dimethyl-5-pentylfuran-2(5H)-one, ginnalin C, 2,3-dioxy-3-O-butenoyloxy-L-galate, 9-methoxyquin-6-one, and 3-O-(3-O-methylgalloyl) quinic acid, continuously accumulated (Figure 5). Combined K-means clustering and heatmap analyses revealed three variation patterns: metabolites that gradually accumulated (mostly flavonoids), metabolites that progressively decreased (some lipids and terpenoids), and metabolites peaking at ZM2012, showing an “increase–decrease” trend. These results were consistent with the volcano plot analysis and collectively indicated that Liupao tea undergoes substantial metabolic reprogramming during the intermediate aging stage.

### 3.5. Correlation Analysis of Sensory Characteristics and Targeted Metabolites in Liupao Tea with Different Aging Durations

**Figure 6:**
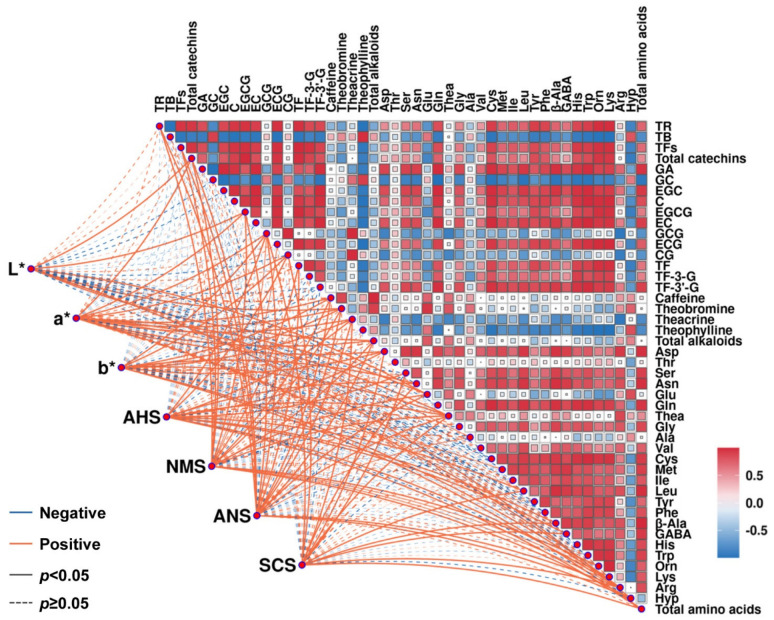
Correlation network analysis of sensory attributes and targeted metabolites in liupao tea across different aging durations.

To elucidate the relationships between sensory quality and chemical composition during Liupao tea aging, correlation analysis was conducted among electronic tongue responses, color parameters, and targeted metabolites (Table S1 in the Supplementary Materials and Figure 6). For taste attributes, metabolites with strong correlations (r ≥ 0.8, *p* < 0.05) were selected. The results showed that sourness (AHS) was positively correlated with gallocatechin gallate (GCG), catechin gallate (CG), theacrine, arginine, aspartic acid, and theanine. Umami (NMS) and sweetness (ANS) were significantly associated with theobromine and glutamic acid. Bitterness (SCS) was also positively associated with GCG, CG, theacrine, arginine, aspartic acid, and theanine. For color attributes, metabolites with moderate to strong correlations (r ≥ 0.6, *p* < 0.05) were identified. L* value was positively correlated with theobromine and glutamic acid, while a* value was positively correlated with theobromine, glutamic acid, and theanine. b* value showed significant positive correlations with GCG, CG, theanine, arginine, and aspartic acid (*p* < 0.05). Overall, theobromine and glutamic acid were identified as key metabolites associated with umami, sweetness, brightness, and redness, whereas theanine was closely related to redness and yellowness. In contrast, GCG, CG, theacrine, aspartic acid, and arginine were mainly associated with bitterness and yellowness. These results demonstrate that coordinated changes in metabolite accumulation and degradation jointly regulate the sensory attributes of Liupao tea during aging.

### 3.6. Correlation Analysis of Sensory Characteristics and Untargeted Metabolites in Liupao Tea with Different Aging Durations

**Figure 7:**
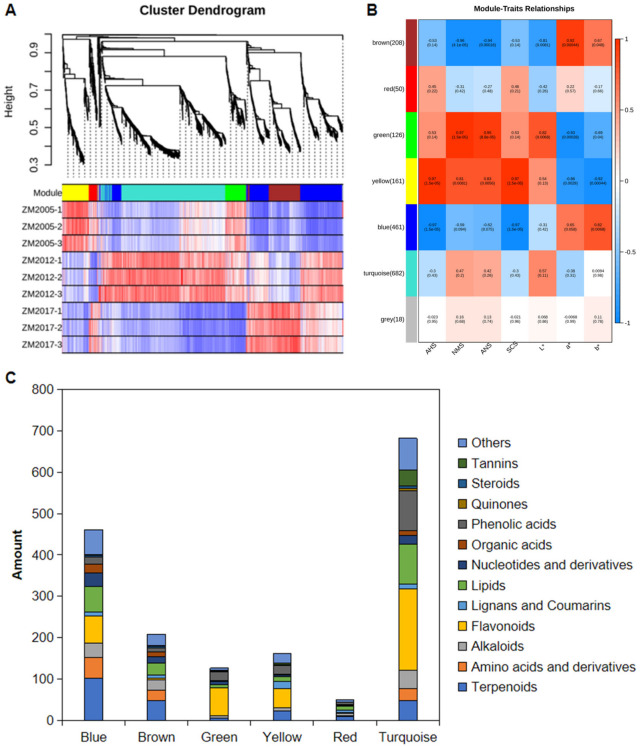
Weighted gene co-expression network analysis (WGCNA)-based co-expression module analysis of metabolites in liupao tea. (**A**) Hierarchical clustering dendrogram of metabolites and module assignment. (**B**) Distribution of different metabolite classes in key modules. (**C**) Distribution of metabolite types across different modules.

To explore the link between metabolites and sensory quality during Liupao tea aging, WGCNA was applied to widely targeted metabolomic data (Figure 7A). All identified metabolites were grouped into seven modules: blue (461), red (50), brown (208), yellow (161), green (126), turquoise (682), and gray (18) (Figure 7B). Module–trait analysis revealed distinct correlations with tea color and electronic tongue responses. The brown module correlated negatively with L*, umami (NMS), and sweetness (ANS), but positively with a* and b*. The green module showed the opposite trend, positively correlating with L*, NMS, and ANS, and negatively with a* and b*. The yellow module was positively associated with all taste attributes and negatively with a* and b*, while the blue module correlated negatively with sourness (AHS) and bitterness (SCS) but positively with a* and b*. Red, turquoise, and gray modules showed no significant sensory correlations. The four key modules (blue, brown, green, yellow) contained diverse metabolites, including terpenoids, amino acids, alkaloids, flavonoids, lignans/coumarins, lipids, nucleotides, organic acids, phenolic acids, quinones, steroids, tannins, and others (Figure 7B,C). Electronic tongue responses were positively linked to the yellow (flavonoids/phenolic acids) and green (flavonoids/terpenoids) modules, but negatively linked to the brown and blue modules. L* value correlated positively with the green module and negatively with the brown module, suggesting that flavonoids and terpenoids may be associated with tea liquor brightness. Conversely, a* and b* values positively correlated with brown and blue modules but negatively with green and yellow, indicating flavonoids, terpenoids, and phenolic acids possibly influence the color of Liupao tea infusion. Overall, these results show that metabolite accumulation patterns are closely linked to the development of tea color and taste during Liupao tea aging.

## 4. Discussion

### 4.1. Dynamic Changes in Taste and Color of Liupao Tea and Their Responses to Aging Time Based on Electronic Tongue and Colorimeter

The use of electronic tongue and colorimeter enables objective tracking of taste and color changes in Liupao tea during aging, accurately revealing sensory differences among samples of different ages [3,13,14]. In terms of taste, electronic tongue results showed that the longest-aged sample (ZM2005) exhibited the highest sweetness but the lowest sourness, umami, and bitterness, whereas the shortest-aged sample (ZM2017) had the strongest umami but the lowest sweetness (Figure 1A,B). This aligns with previous studies showing that long-term dark tea aging leads to decreases in sourness and bitterness, while an increase in the desirable attributes such as sweetness [24,25,26]. Moreover, the electronic tongue results suggest that taste evolution is not a simple linear decline; for instance, tea aged longer, such as over 15 years, may exhibit a richer flavor from accumulated sweetness. However, the taste attributes in this study refer to sensor responses rather than verified human sensory evaluations. Further validation of these findings is needed in future studies. Regarding color, this study found that tea liquor brightness (L*) generally increased with aging, although its trend varied depending on brewing conditions, while redness (a*) and yellowness (b*) significantly decreased (*p* < 0.05) (Figure 1C). This differs from previous reports describing a shift toward a reddish color [6,27], possibly due to the longer aging durations (over 15 years) investigated in this study. Correlation analysis further revealed that L* was positively correlated with umami (NMS) and sweetness (ANS), whereas a* and b* were negatively correlated with most taste responses (Figure 1D). With prolonged storage, the decrease in bitter and sour compounds and the increase in polysaccharides reduce bitterness, sourness, and umami while enhancing sweetness, resulting in higher SCS, AHS, NMS, and ANS values (Figure 1A) [14,28]. Tea liquor brightness is positively correlated with catechins and negatively correlated with amino acids [18]. The increase in catechins and decrease in amino acids during storage may enhance the brightness of Liubao tea liquor, resulting in a higher L* value (Figure 1C). These findings statistically indicate an intrinsic synergistic or antagonistic relationship between color darkening and taste transformation during aging [12]. However, most studies, including the present study, focus on macroscopic trends and correlations [6,7,9], while the molecular mechanisms by which aging affects biochemical pathways and sensory changes remain unclear. In addition, developing predictive models to quantify sensory traits or determine optimal aging is a key future direction.

### 4.2. Temporal Evolution Patterns of Quality-Related Metabolites in Liupao Tea Based on Metabolomics

The integration of targeted and untargeted metabolomics provides a powerful approach for systematically elucidating the reconstruction of non-volatile metabolites during Liupao tea aging [20]. Targeted metabolite analysis in this study clearly characterized the dynamic patterns of three key metabolite classes: catechins and total free amino acids, both of which peaked at the intermediate aging stage (ZM2012), exhibiting a non-linear “accumulation–degradation” trend (Table 1). In contrast, alkaloids showed compound-specific responses, with caffeine remaining the most stable and theobromine reaching its highest level in the shortest-aged sample (ZM2017). This “mid-stage peak” phenomenon suggests that aging is not a simple degradation process but involves an active phase of metabolite transformation and synthesis [7]. Untargeted metabolomics further confirmed systematic changes in metabolic profiles from a global perspective. The OPLS-DA model showed clear separation among groups and good model reliability (Figure 2) [29]. In the study, among 1897 metabolites, flavonoids increased with aging (24.4% in ZM2017 to 49.9% in ZM2005), while terpenoids and lipids decreased. Furthermore, volcano plots showed the most differential metabolites at ZM2012, marking it as a key stage for metabolic reprogramming (Figure 4 and Figure 5). These findings are consistent with previous reports on catechin oxidation and flavonoid modification pathways [7,15], while further demonstrating that metabolite changes are not monotonic but exhibit clear stage-dependent dynamics. However, most studies, including the present work, mainly focus on describing differential metabolites and their variation trends [7,16], while the upstream regulators (e.g., microbial genes, enzyme activities) and their downstream metabolic interactions remain poorly understood [30]. In addition, although three aging time points were investigated, a more densely sampled aging time-series is still needed to more precisely capture the kinetics of metabolite transformations.

### 4.3. Linking Sensory Traits and Metabolites to Identify Key Flavor Metabolites in Liupao Tea

Integrating sensory data from the electronic tongue and colorimeter with metabolomics is a key strategy for uncovering the material basis of Liupao tea flavor [12,13,14]. In this study, correlation network and WGCNA analyses were used to construct a preliminary “sensory attribute–metabolite” map (Figure 6 and Figure 7). Correlation analysis showed that tea liquor brightness (L*) positively correlated with theobromine, while redness (a*) correlated positively with most metabolites. For taste, umami (NMS) and sweetness (ANS) correlated positively with multiple metabolite classes, whereas sourness (AHS) and bitterness (SCS) correlated negatively with TFs and catechins but positively with alkaloids and amino acids. These associations suggest that brightness may relate to preserved antioxidant polyphenols, while changes in red and yellow hues may be associated with accumulated oxidation products such as TBs [12]. WGCNA grouped metabolites into seven co-expression modules, with the brown, green, yellow, and blue modules showing significant correlations with sensory traits (*p* < 0.05). These modules were enriched in terpenoids, amino acid derivatives, alkaloids, flavonoids, and lipids, indicating that color and taste arise from the synergistic effects of multiple metabolites (Figure 7). For example, the green module, linked to L* and sweet/umami taste, and the blue module, linked to a* and b*, had distinct metabolite compositions, potentially representing two chemical clusters controlling color and taste, respectively. However, current analyses, including this study, focus on statistical correlations without revealing causal biological mechanisms. Moreover, this study did not integrate microbiome data, leaving the full chain of “aging time–microbial succession–metabolite transformation–sensory quality” unresolved [7,26]. Future studies should include finer aging time points and multi-omics integration to uncover the core networks controlling Liupao tea flavor.

### 4.4. Limitations and Future Perspectives

Although this study systematically investigated the relationships between sensory characteristics and non-volatile metabolites during the aging of Liupao tea, several limitations should be acknowledged. This study mainly focused on statistical correlations between metabolites and sensory traits, while the underlying causal mechanisms remain unclear. In particular, microbiological data were not included, making it difficult to identify the upstream regulatory mechanisms responsible for the observed metabolic changes. Previous studies on dark tea and Pu-erh tea have suggested that microbial succession during aging may play an important role in regulating metabolite transformation and flavor formation. Dominant microorganisms such as Aspergillus, Bacillus, and yeasts can secrete extracellular enzymes, including polyphenol oxidases and glycosidases, thereby promoting catechin oxidation, flavonoid modification, polysaccharide degradation, and the formation of TBs during aging [31,32]. In addition, microbial metabolism may influence the conversion of amino acids, organic acids, and phenolic compounds, ultimately affecting the sweetness, bitterness, umami, and color characteristics of aged tea infusions [32,33]. In addition, microbial regulation and volatile compounds related to aged aroma were not investigated. Furthermore, only three aging time points (8, 13, and 20 years) were investigated, which may not fully capture the continuous dynamic changes occurring during long-term aging. More aging intervals and multi-omics analyses are needed to better understand metabolite transformation and flavor formation in Liupao tea.

## 5. Conclusions

This study systematically revealed how aging affects Liupao tea’s sensory quality and non-volatile metabolites by integrating intelligent sensory analysis with multi-omics approaches. Aging drives coordinated changes in taste, color, and chemical composition. The longest-aged sample (ZM2005) had the highest sweetness but lowest sourness, umami, and bitterness, while the shortest-aged sample (ZM2017) showed the strongest umami and lowest sweetness. Tea liquor brightness (L*) generally increased with aging, whereas redness (a*) and yellowness (b*) decreased, with color changes linked to specific taste attributes. Metabolically, aging represents a dynamic transformation network rather than simple degradation. At the intermediate stage (ZM2012), catechins and free amino acids peaked in an “accumulation–degradation” pattern, marking a critical turning point of metabolic reprogramming. Correlation analysis identified key metabolites, including theobromine, glutamic acid, and theanine, associated with umami, sweetness, and liquor color, as well as GCG, theacrine, aspartic acid, and arginine linked to bitterness and yellowness. WGCNA-based “sensory–metabolite” networks showed flavonoid- and terpenoid-enriched modules jointly regulate color and taste. However, all samples are from a single producer and may not be representative of Liupao tea produced under different conditions and by different producers. Overall, these findings provide the chemical basis of Liupao tea’s quality and provide a foundation for quality-directed aging optimization.

## Figures and Tables

**Table 1:** Targeted analysis of non-volatile metabolites in Liupao tea with different aging durations.

Compound	ZM2005	ZM2012	ZM2017
TR *	2.0112 ± 0.1454 b	3.5221 ± 0.2170 a	1.0868 ± 0.1937 c
TB *	15.1410 ± 0.4468 b	10.9523 ± 0.5451 c	16.4784 ± 0.3111 a
TFs	0.0659 ± 0.0015 b	0.0837 ± 0.0007 a	0.0544 ± 0.0046 c
Total catechins	5.2093 ± 0.4415 b	7.6979 ± 0.4609 a	1.9676 ± 0.1149 c
GA	0.0887 ± 0.0168 c	4.1955 ± 0.3734 a	0.6943 ± 0.0235 b
GC	1.2286 ± 0.0350 a	0.2051 ± 0.0147 c	1.0531 ± 0.1140 b
EGC	0 ± 0 b	0.2858 ± 0.0320 a	0.0219 ± 0.0099 b
C	0.1866 ± 0.0087 b	0.8668 ± 0.0697 a	0.0406 ± 0.0026 c
EGCG	1.2678 ± 0.0632 b	2.3529 ± 0.1611 a	0.1973 ± 0.0019 c
EC	0.2598 ± 0.0031 b	1.8684 ± 0.1058 a	0.2162 ± 0.0069 b
GCG	0.6022 ± 0.0982 a	0.0191 ± 0.0021 b	0.0111 ± 0.0004 b
ECG	0.8908 ± 0.0514 b	2.0587 ± 0.1076 a	0.3741 ± 0.0169 c
CG	0.7734 ± 0.2314 a	0.0411 ± 0.0013 b	0.0532 ± 0.0007 b
TF	0.0336 ± 0.0010 b	0.0430 ± 0.0002 a	0.0256 ± 0.0014 c
TF-3-G	0.0257 ± 0.0007 b	0.0301 ± 0.0001 a	0.0221 ± 0.0031 b
TF-3′-G	0.0066 ± 0.0002 b	0.0106 ± 0.0008 a	0.0066 ± 0.0003 b
CAF	42.2122 ± 3.876 a	44.0087 ± 3.4669 a	48.6387 ± 0.2082 a
Theobromine	1.3777 ± 0.1394 b	1.5298 ± 0.1355 b	2.2363 ± 0.0442 a
Theacrine	0.0237 ± 0.0050 a	0.0005 ± 0.0001 b	0.0030 ± 0.0001 b
Theophylline	0.9025 ± 0.0547 b	0.2347 ± 0.0240 c	1.1072 ± 0.0260 a
Total alkaloids	44.5162 ± 4.0708 a	45.7736 ± 3.6241 a	51.9851 ± 0.2303 a
Asp	0.0089 ± 0.0008 c	0.0291 ± 0.0005 a	0.0266 ± 0.0008 b
Thr	0.0070 ± 0.0016 a	0.0072 ± 0.0003 a	0.0070 ± 0.0002 a
Ser	0.0038 ± 0.0005 b	0.0053 ± 0.0003 a	0.0051 ± 0.0001 b
Asn	0.0278 ± 0.0088 c	0.0741 ± 0.0031 a	0.0650 ± 0.0028 b
Glu	0.0072 ± 0.0000 b	0.0072 ± 0.0001 b	0.0078 ± 0.0002 a
Gln	0.0077 ± 0.0001 c	0.0296 ± 0.0013 a	0.0237 ± 0.0016 b
Thea	0.0389 ± 0.0016 c	0.0627 ± 0.0023 b	0.0646 ± 0.0071 a
Gly	0.0041 ± 0.0000 b	0.0063 ± 0.0010 a	0.006 ± 0.0004 ab
Ala	0.0080 ± 0.0001 a	0.0082 ± 0.0001 a	0.0084 ± 0.0009 a
Val	0.0067 ± 0.0000 a	0.0072 ± 0.0002 a	0.0071 ± 0.0003 a
Cys	0.0235 ± 0.0002 b	0.0410 ± 0.0009 a	0.0351 ± 0.0008 b
Met	0.0194 ± 0.0000 b	0.0254 ± 0.0006 a	0.0250 ± 0.0025 b
Ile	0.0126 ± 0.0000 b	0.0139 ± 0.0004 a	0.0138 ± 0.0007 b
Leu	0.0131 ± 0.0001 c	0.0180 ± 0.0005 a	0.0168 ± 0.0005 b
Tyr	0.0265 ± 0.0001 b	0.0321 ± 0.0005 a	0.0304 ± 0.0036 b
Phe	0.0119 ± 0.0011 b	0.0163 ± 0.0001 a	0.0147 ± 0.0020 b
β-Ala	0.0027 ± 0.0003 c	0.0090 ± 0.0004 a	0.0079 ± 0.0003 b
GABA	0.0075 ± 0.0003 b	0.0296 ± 0.0041 a	0.0267 ± 0.0069 b
His	0.0010 ± 0.0001 b	0.0024 ± 0.0001 a	0.0020 ± 0.0004 b
Trp	0.0053 ± 0.0000 b	0.0230 ± 0.0019 a	0.0173 ± 0.0005 b
Orn	0.0110 ± 0.0005 b	0.0211 ± 0.0005 a	0.0158 ± 0.0007 c
Lys	0.0024 ± 0.0000 b	0.0063 ± 0.0001 a	0.0044 ± 0.0000 c
Arg	0.0070 ± 0.0002 b	0.0136 ± 0.0004 a	0.0132 ± 0.0010 a
Hyp	0.0009 ± 0.0002 b	0.0006 ± 0.0001 b	0.0008 ± 0.0001 a
Total amino acids	0.2651 ± 0.0055 c	0.4891 ± 0.0017 a	0.3495 ± 0.0130 b

* indicates that the unit for this compound is %, whereas the units for all other compounds are mg/g. Different letters indicate significant differences among samples (*p* < 0.05). Ala, alanine; Arg, arginine; Asn, asparagine; Asp, aspartic acid; C, catechin; CAF, caffeine; CG, catechin gallate; Cys, cystine; EC, epicatechin; ECG, epicatechin gallate; EGCG, epigallocatechin gallate; EGC, epigallocatechin; GA, gallic acid; GABA, γ-aminobutyric acid; GC, gallocatechin; GCG, gallocatechin gallate; Glu, glutamic acid; Gln, glutamine; Gly, glycine; His, histidine; Hyp, hydroxyproline; Ile, isoleucine; Leu, leucine; Lys, lysine; Met, methionine; Orn, ornithine; Phe, phenylalanine; Ser, serine; TB, theabrownin; TF, theaflavin; TF-3-G, theaflavin-3-gallate; TF-3’-G, theaflavin-3’-gallate; TFs, theaflavins; Thea, theanine; Thr, threonine; TR, thearubigins; Trp, tryptophan; Tyr, tyrosine; Val, valine; β-Ala, β-alanine.

## Data Availability

Data Availability Statement: The original contributions presented in the study are included in the article/Supplementary Material, further inquiries can be directed to the corresponding authors.

## Supplementary Materials

The following supporting information can be downloaded at: https://www.mdpi.com/article/10.3390/foods15111851/s1. Table S1: Correlation between sensory attributes and targeted metabolites in Liupao tea with different aging durations; Figure S1: Difference analysis of taste and color changes in Liupao tea infusions with different aging durations; Figure S2: Chromatograms of standards and typical tea sample; Figure S3: Coefficient of variation analysis of metabolites.

## Abbreviations

The following abbreviations are used in this manuscript:

CG	Catechin gallate
CV	Coefficient of variation
GCG	Gallocatechin gallate
OPLS-DA	Orthogonal partial least squares discriminant analysis
TR	Thearubigin
TB	Theabrownin
TF	Theaflavin
VIP	Variable importance in projection
WGCNA	Weighted gene co-expression network analysis
