# Supplementary material for: Sensory and Metabolomic Analysis Reveal the Quality Evolution of Liupao Tea During Long-Term Aging
Source: Foods. 2026 May 23;15(11):1851. doi: 10.3390/foods15111851 (PMC13256477; doi:10.3390/foods15111851)
Supplement: Supplementary file 1 [file foods-15-01851-s001.zip › foods-4286028-supplementary.pdf]

# Supplementary Materials

**Table S1** Correlation between sensory attributes and targeted metabolites in Liupao tea with different aging durations.

| Sensory attribute | Targeted metabolite | r | p | r.sign |
|---|---|---|---|---|
| L* | TR | 0.22382881 | 0.1 | Positive |
| L* | TB | 0.00605433 | 0.396 | Positive |
| L* | TFs | 0.14351455 | 0.18 | Positive |
| L* | Total catechins | 0.43899424 | 0.021 | Positive |
| L* | GA | -0.0896112 | 0.634 | Negative |
| L* | GC | -0.1316913 | 0.772 | Negative |
| L* | EGC | -0.121916 | 0.732 | Negative |
| L* | C | 0.03346812 | 0.337 | Positive |
| L* | EGCG | 0.36977044 | 0.034 | Positive |
| L* | EC | -0.066583 | 0.581 | Negative |
| L* | GCG | -0.0600131 | 0.51 | Negative |
| L* | ECG | 0.16842815 | 0.145 | Positive |
| L* | CG | -0.1370878 | 0.68 | Negative |
| L* | TF | 0.26135022 | 0.07 | Positive |
| L* | TF-3-G | 0.03586052 | 0.419 | Positive |
| L* | TF-3'-G | -0.1094875 | 0.661 | Negative |
| L* | Caffeine | 0.41639905 | 0.051 | Positive |
| L* | Theobromine | 0.64103198 | 0.002 | Positive |
| L* | Theacrine | -0.0843951 | 0.559 | Negative |
| L* | Theophylline | 0.13209695 | 0.202 | Positive |
| L* | Total alkaloids | 0.48486355 | 0.023 | Positive |
| L* | Asp | -0.0872008 | 0.684 | Negative |
| L* | Thr | -0.0466622 | 0.532 | Negative |
| L* | Ser | -0.2109994 | 0.827 | Negative |
| L* | Asn | -0.1181519 | 0.701 | Negative |
| L* | Glu | 0.66674644 | 0.001 | Positive |
| L* | Gln | -0.1194772 | 0.729 | Negative |
| L* | Thea | 0.04720237 | 0.36 | Positive |
| L* | Gly | -0.0371802 | 0.459 | Negative |
| L* | Ala | 0.06642316 | 0.373 | Positive |
| L* | Val | -0.2261824 | 0.949 | Negative |
| L* | Cys | -0.0979606 | 0.672 | Negative |
| L* | Met | -0.1758765 | 0.95 | Negative |
| L* | Ile | -0.040181 | 0.508 | Negative |
| L* | Leu | -0.14819 | 0.823 | Negative |
| L* | Tyr | 0.00308229 | 0.483 | Positive |
| L* | Phe | -0.0217575 | 0.507 | Negative |
| L* | β-Ala | -0.0918809 | 0.661 | Negative |
| L* | GABA | -0.1841689 | 0.924 | Negative |

| | | | | |
|---|---|---|---|---|
| L* | His | -0.1446706 | 0.797 | Negative |
| L* | Trp | -0.0372099 | 0.489 | Negative |
| L* | Orn | 0.20078125 | 0.116 | Positive |
| L* | Lys | 0.15927228 | 0.147 | Positive |
| L* | Arg | -0.0730309 | 0.589 | Negative |
| L* | Hyp | 0.43518568 | 0.02 | Positive |
| L* | Total amino acids | -0.0954248 | 0.684 | Negative |
| a* | TR | 0.209397 | 0.096 | Positive |
| a* | TB | 0.05122353 | 0.304 | Positive |
| a* | TFs | 0.23911527 | 0.087 | Positive |
| a* | Total catechins | 0.45485284 | 0.035 | Positive |
| a* | GA | -0.0133254 | 0.433 | Negative |
| a* | GC | -0.0329731 | 0.482 | Negative |
| a* | EGC | -0.083512 | 0.618 | Negative |
| a* | C | 0.0269518 | 0.363 | Positive |
| a* | EGCG | 0.4005891 | 0.044 | Positive |
| a* | EC | -0.089093 | 0.619 | Negative |
| a* | GCG | 0.54775726 | 0.021 | Positive |
| a* | ECG | 0.16715222 | 0.127 | Positive |
| a* | CG | 0.44382797 | 0.027 | Positive |
| a* | TF | 0.34400033 | 0.051 | Positive |
| a* | TF-3-G | 0.19086034 | 0.092 | Positive |
| a* | TF-3'-G | -0.1328921 | 0.789 | Negative |
| a* | Caffeine | 0.41795325 | 0.02 | Positive |
| a* | Theobromine | 0.74227609 | 0.003 | Positive |
| a* | Theacrine | 0.49178243 | 0.027 | Positive |
| a* | Theophylline | 0.07514212 | 0.255 | Positive |
| a* | Total alkaloids | 0.47091863 | 0.014 | Positive |
| a* | Asp | 0.41484695 | 0.027 | Positive |
| a* | Thr | 0.06291882 | 0.32 | Positive |
| a* | Ser | 0.08829137 | 0.244 | Positive |
| a* | Asn | 0.16890395 | 0.119 | Positive |
| a* | Glu | 0.65938255 | 0.008 | Positive |
| a* | Gln | -0.0379042 | 0.501 | Negative |
| a* | Thea | 0.74550788 | 0.003 | Positive |
| a* | Gly | 0.05354893 | 0.305 | Positive |
| a* | Ala | 0.28345228 | 0.029 | Positive |
| a* | Val | 0.01355845 | 0.347 | Positive |
| a* | Cys | -0.1039092 | 0.66 | Negative |
| a* | Met | -0.0460655 | 0.511 | Negative |
| a* | Ile | -0.0600951 | 0.57 | Negative |
| a* | Leu | 0.14637753 | 0.155 | Positive |
| a* | Tyr | 0.0226758 | 0.386 | Positive |
| a* | Phe | -0.1053436 | 0.727 | Negative |

| | | | | |
|---|---|---|---|---|
| a* | β-Ala | 0.1517543 | 0.137 | Positive |
| a* | GABA | 0.08975712 | 0.206 | Positive |
| a* | His | -0.0675498 | 0.586 | Negative |
| a* | Trp | -0.0845654 | 0.614 | Negative |
| a* | Orn | 0.16764198 | 0.136 | Positive |
| a* | Lys | 0.16957362 | 0.127 | Positive |
| a* | Arg | 0.5313339 | 0.022 | Positive |
| a* | Hyp | 0.33930186 | 0.054 | Positive |
| a* | Total amino acids | 0.1992363 | 0.102 | Positive |
| b* | TR | -0.0405119 | 0.534 | Negative |
| b* | TB | -0.0961938 | 0.659 | Negative |
| b* | TFs | -0.0068832 | 0.46 | Negative |
| b* | Total catechins | 0.01395539 | 0.416 | Positive |
| b* | GA | 0.00716452 | 0.35 | Positive |
| b* | GC | 0.00254464 | 0.399 | Positive |
| b* | EGC | -0.0816558 | 0.58 | Negative |
| b* | C | -0.0997245 | 0.655 | Negative |
| b* | EGCG | 0.01434808 | 0.394 | Positive |
| b* | EC | -0.1169154 | 0.724 | Negative |
| b* | GCG | 0.74550079 | 0.007 | Positive |
| b* | ECG | -0.0620624 | 0.542 | Negative |
| b* | CG | 0.63729453 | 0.008 | Positive |
| b* | TF | 0.02584559 | 0.365 | Positive |
| b* | TF-3-G | -0.0055788 | 0.447 | Negative |
| b* | TF-3'-G | -0.1493019 | 0.792 | Negative |
| b* | Caffeine | 0.37112774 | 0.043 | Positive |
| b* | Theobromine | 0.31653747 | 0.043 | Positive |
| b* | Theacrine | 0.69464825 | 0.016 | Positive |
| b* | Theophylline | -0.0806735 | 0.632 | Negative |
| b* | Total alkaloids | 0.3320818 | 0.06 | Positive |
| b* | Asp | 0.58447362 | 0.014 | Positive |
| b* | Thr | 0.00284539 | 0.449 | Positive |
| b* | Ser | 0.20002937 | 0.146 | Positive |
| b* | Asn | 0.3382247 | 0.036 | Positive |
| b* | Glu | 0.11695005 | 0.228 | Positive |
| b* | Gln | -0.012721 | 0.385 | Negative |
| b* | Thea | 0.79372326 | 0.002 | Positive |
| b* | Gly | 0.12830978 | 0.19 | Positive |
| b* | Ala | 0.13805783 | 0.243 | Positive |
| b* | Val | 0.22269121 | 0.111 | Positive |
| b* | Cys | -0.1018503 | 0.69 | Negative |
| b* | Met | 0.03460667 | 0.328 | Positive |
| b* | Ile | -0.0780482 | 0.615 | Negative |
| b* | Leu | 0.28744054 | 0.068 | Positive |

| | | | | |
|---|---|---|---|---|
| b* | Tyr | -0.1230091 | 0.711 | Negative |
| b* | Phe | -0.1597587 | 0.824 | Negative |
| b* | β-Ala | 0.24580052 | 0.058 | Positive |
| b* | GABA | 0.28536926 | 0.067 | Positive |
| b* | His | -0.0841093 | 0.632 | Negative |
| b* | Trp | -0.1318959 | 0.759 | Negative |
| b* | Orn | -0.0657611 | 0.562 | Negative |
| b* | Lys | -0.0398721 | 0.49 | Negative |
| b* | Arg | 0.74214872 | 0.013 | Positive |
| b* | Hyp | -0.0764806 | 0.637 | Negative |
| b* | Total amino acids | 0.30735476 | 0.041 | Positive |
| AHS | TR | -0.0640892 | 0.537 | Negative |
| AHS | TB | -0.0456208 | 0.452 | Negative |
| AHS | TFs | -0.0610913 | 0.578 | Negative |
| AHS | Total catechins | -0.0671582 | 0.547 | Negative |
| AHS | GA | 0.1819416 | 0.179 | Positive |
| AHS | GC | 0.18992116 | 0.128 | Positive |
| AHS | EGC | 0.0841621 | 0.215 | Positive |
| AHS | C | -0.0007592 | 0.343 | Negative |
| AHS | EGCG | -0.0351459 | 0.485 | Negative |
| AHS | EC | 0.02112241 | 0.293 | Positive |
| AHS | GCG | 0.95426754 | 0.007 | Positive |
| AHS | ECG | -0.0121852 | 0.414 | Negative |
| AHS | CG | 0.87717709 | 0.004 | Positive |
| AHS | TF | -0.0453352 | 0.505 | Negative |
| AHS | TF-3-G | -0.1341106 | 0.676 | Negative |
| AHS | TF-3'-G | 0.00247536 | 0.291 | Positive |
| AHS | Caffeine | 0.24258913 | 0.08 | Positive |
| AHS | Theobromine | 0.1396664 | 0.198 | Positive |
| AHS | Theacrine | 0.93592237 | 0.003 | Positive |
| AHS | Theophylline | -0.0063399 | 0.378 | Negative |
| AHS | Total alkaloids | 0.15131403 | 0.101 | Positive |
| AHS | Asp | 0.81276011 | 0.003 | Positive |
| AHS | Thr | 0.21360386 | 0.161 | Positive |
| AHS | Ser | 0.35977165 | 0.044 | Positive |
| AHS | Asn | 0.60618429 | 0.01 | Positive |
| AHS | Glu | -0.0483632 | 0.439 | Negative |
| AHS | Gln | 0.16479188 | 0.197 | Positive |
| AHS | Thea | 0.8068982 | 0.013 | Positive |
| AHS | Gly | 0.33683964 | 0.058 | Positive |
| AHS | Ala | -0.1306174 | 0.698 | Negative |
| AHS | Val | 0.26667903 | 0.105 | Positive |
| AHS | Cys | 0.03263663 | 0.279 | Positive |
| AHS | Met | 0.29983598 | 0.04 | Positive |

| | | | | |
|---|---|---|---|---|
| AHS | Ile | 0.10745321 | 0.23 | Positive |
| AHS | Leu | 0.46433033 | 0.01 | Positive |
| AHS | Tyr | -0.0923147 | 0.546 | Negative |
| AHS | Phe | -0.0063062 | 0.433 | Negative |
| AHS | β-Ala | 0.48751909 | 0.01 | Positive |
| AHS | GABA | 0.51670593 | 0.033 | Positive |
| AHS | His | -0.006957 | 0.392 | Negative |
| AHS | Trp | 0.00820188 | 0.273 | Positive |
| AHS | Orn | -0.0158314 | 0.437 | Negative |
| AHS | Lys | 0.01193803 | 0.363 | Positive |
| AHS | Arg | 0.93472278 | 0.006 | Positive |
| AHS | Hyp | -0.1543872 | 0.838 | Negative |
| AHS | Total amino acids | 0.53683663 | 0.016 | Positive |
| NMS | TR | 0.35927881 | 0.035 | Positive |
| NMS | TB | 0.14164716 | 0.19 | Positive |
| NMS | TFs | 0.35657291 | 0.029 | Positive |
| NMS | Total catechins | 0.65471735 | 0.016 | Positive |
| NMS | GA | -0.0443695 | 0.477 | Negative |
| NMS | GC | -0.0585533 | 0.512 | Negative |
| NMS | EGC | -0.0836023 | 0.578 | Negative |
| NMS | C | 0.09459102 | 0.263 | Positive |
| NMS | EGCG | 0.57292826 | 0.026 | Positive |
| NMS | EC | -0.0627034 | 0.504 | Negative |
| NMS | GCG | 0.29213038 | 0.022 | Positive |
| NMS | ECG | 0.28259834 | 0.037 | Positive |
| NMS | CG | 0.21071442 | 0.123 | Positive |
| NMS | TF | 0.49755347 | 0.026 | Positive |
| NMS | TF-3-G | 0.28775835 | 0.063 | Positive |
| NMS | TF-3'-G | -0.1275498 | 0.759 | Negative |
| NMS | Caffeine | 0.47594598 | 0.026 | Positive |
| NMS | Theobromine | 0.94473366 | 0.001 | Positive |
| NMS | Theacrine | 0.2531162 | 0.055 | Positive |
| NMS | Theophylline | 0.17973913 | 0.136 | Positive |
| NMS | Total alkaloids | 0.57478334 | 0.01 | Positive |
| NMS | Asp | 0.18416356 | 0.086 | Positive |
| NMS | Thr | 0.02824738 | 0.405 | Positive |
| NMS | Ser | -0.1126514 | 0.715 | Negative |
| NMS | Asn | 0.05719334 | 0.313 | Positive |
| NMS | Glu | 0.90722718 | 0.001 | Positive |
| NMS | Gln | -0.0570976 | 0.487 | Negative |
| NMS | Thea | 0.54058444 | 0.016 | Positive |
| NMS | Gly | -0.1039729 | 0.612 | Negative |
| NMS | Ala | 0.3331475 | 0.055 | Positive |
| NMS | Val | -0.0919312 | 0.661 | Negative |

| | | | | |
|---|---|---|---|---|
| NMS | Cys | -0.0806038 | 0.566 | Negative |
| NMS | Met | -0.0880112 | 0.647 | Negative |
| NMS | Ile | -0.0578223 | 0.549 | Negative |
| NMS | Leu | 0.01794472 | 0.378 | Positive |
| NMS | Tyr | 0.1436541 | 0.209 | Positive |
| NMS | Phe | 0.01847483 | 0.381 | Positive |
| NMS | β-Ala | 0.04677886 | 0.327 | Positive |
| NMS | GABA | -0.0375057 | 0.473 | Negative |
| NMS | His | -0.0543539 | 0.537 | Negative |
| NMS | Trp | -0.0473134 | 0.459 | Negative |
| NMS | Orn | 0.30524962 | 0.035 | Positive |
| NMS | Lys | 0.281178 | 0.037 | Positive |
| NMS | Arg | 0.25241581 | 0.047 | Positive |
| NMS | Hyp | 0.5262592 | 0.027 | Positive |
| NMS | Total amino acids | 0.08226452 | 0.276 | Positive |
| ANS | TR | 0.32577811 | 0.039 | Positive |
| ANS | TB | 0.11653454 | 0.212 | Positive |
| ANS | TFs | 0.31518413 | 0.046 | Positive |
| ANS | Total catechins | 0.59151578 | 0.015 | Positive |
| ANS | GA | -0.0569869 | 0.496 | Negative |
| ANS | GC | -0.0720792 | 0.544 | Negative |
| ANS | EGC | -0.1027891 | 0.658 | Negative |
| ANS | C | 0.05568919 | 0.333 | Positive |
| ANS | EGCG | 0.51207762 | 0.022 | Positive |
| ANS | EC | -0.092443 | 0.606 | Negative |
| ANS | GCG | 0.37424349 | 0.014 | Positive |
| ANS | ECG | 0.23037567 | 0.075 | Positive |
| ANS | CG | 0.28776717 | 0.063 | Positive |
| ANS | TF | 0.45293822 | 0.028 | Positive |
| ANS | TF-3-G | 0.24088318 | 0.084 | Positive |
| ANS | TF-3'-G | -0.1456613 | 0.822 | Negative |
| ANS | Caffeine | 0.44857914 | 0.036 | Positive |
| ANS | Theobromine | 0.88016514 | 0.002 | Positive |
| ANS | Theacrine | 0.33399999 | 0.025 | Positive |
| ANS | Theophylline | 0.12355543 | 0.234 | Positive |
| ANS | Total alkaloids | 0.53561818 | 0.011 | Positive |
| ANS | Asp | 0.23489407 | 0.074 | Positive |
| ANS | Thr | 0.04849388 | 0.375 | Positive |
| ANS | Ser | -0.1001736 | 0.701 | Negative |
| ANS | Asn | 0.10054277 | 0.233 | Positive |
| ANS | Glu | 0.80057347 | 0.014 | Positive |
| ANS | Gln | -0.0716645 | 0.545 | Negative |
| ANS | Thea | 0.65101353 | 0.016 | Positive |
| ANS | Gly | -0.0849985 | 0.585 | Negative |

| | | | | |
|---|---|---|---|---|
| ANS | Ala | 0.2997935 | 0.049 | Positive |
| ANS | Val | -0.0282171 | 0.501 | Negative |
| ANS | Cys | -0.1033164 | 0.668 | Negative |
| ANS | Met | -0.0855752 | 0.652 | Negative |
| ANS | Ile | -0.0186882 | 0.477 | Negative |
| ANS | Leu | 0.03231138 | 0.356 | Positive |
| ANS | Tyr | 0.23861771 | 0.109 | Positive |
| ANS | Phe | 0.03622279 | 0.326 | Positive |
| ANS | β-Ala | 0.06413899 | 0.279 | Positive |
| ANS | GABA | -0.0032985 | 0.385 | Negative |
| ANS | His | -0.0672365 | 0.604 | Negative |
| ANS | Trp | -0.0886966 | 0.607 | Negative |
| ANS | Orn | 0.23256704 | 0.077 | Positive |
| ANS | Lys | 0.22889025 | 0.075 | Positive |
| ANS | Arg | 0.33664678 | 0.027 | Positive |
| ANS | Hyp | 0.49086179 | 0.026 | Positive |
| ANS | Total amino acids | 0.10299278 | 0.201 | Positive |
| SCS | TR | -0.0617952 | 0.531 | Negative |
| SCS | TB | -0.0424291 | 0.445 | Negative |
| SCS | TFs | -0.0585477 | 0.571 | Negative |
| SCS | Total catechins | -0.0663611 | 0.522 | Negative |
| SCS | GA | 0.18663443 | 0.133 | Positive |
| SCS | GC | 0.19402768 | 0.126 | Positive |
| SCS | EGC | 0.08832371 | 0.252 | Positive |
| SCS | C | 0.0032219 | 0.331 | Positive |
| SCS | EGCG | -0.0337092 | 0.482 | Negative |
| SCS | EC | 0.02588685 | 0.282 | Positive |
| SCS | GCG | 0.95275124 | 0.007 | Positive |
| SCS | ECG | -0.0089686 | 0.422 | Negative |
| SCS | CG | 0.87507306 | 0.007 | Positive |
| SCS | TF | -0.0434433 | 0.488 | Negative |
| SCS | TF-3-G | -0.1332681 | 0.669 | Negative |
| SCS | TF-3'-G | 0.00731514 | 0.29 | Positive |
| SCS | Caffeine | 0.24685501 | 0.08 | Positive |
| SCS | Theobromine | 0.13779274 | 0.217 | Positive |
| SCS | Theacrine | 0.93499084 | 0.003 | Positive |
| SCS | Theophylline | -0.0029566 | 0.361 | Negative |
| SCS | Total alkaloids | 0.1546304 | 0.144 | Positive |
| SCS | Asp | 0.81447206 | 0.003 | Positive |
| SCS | Thr | 0.21194071 | 0.157 | Positive |
| SCS | Ser | 0.36330158 | 0.038 | Positive |
| SCS | Asn | 0.60919987 | 0.01 | Positive |
| SCS | Glu | -0.052967 | 0.443 | Negative |
| SCS | Gln | 0.16898695 | 0.154 | Positive |

| | | | | |
|---|---|---|---|---|
| SCS | Thea | 0.8053962 | 0.013 | Positive |
| SCS | Gly | 0.34351973 | 0.053 | Positive |
| SCS | Ala | -0.1349412 | 0.72 | Negative |
| SCS | Val | 0.26888174 | 0.103 | Positive |
| SCS | Cys | 0.03692469 | 0.278 | Positive |
| SCS | Met | 0.29932036 | 0.059 | Positive |
| SCS | Ile | 0.11098357 | 0.224 | Positive |
| SCS | Leu | 0.46773324 | 0.016 | Positive |
| SCS | Tyr | -0.0905555 | 0.55 | Negative |
| SCS | Phe | -0.0043055 | 0.433 | Negative |
| SCS | β-Ala | 0.49126227 | 0.016 | Positive |
| SCS | GABA | 0.51660913 | 0.033 | Positive |
| SCS | His | -0.0025295 | 0.38 | Negative |
| SCS | Trp | 0.01272804 | 0.262 | Positive |
| SCS | Orn | -0.0138887 | 0.436 | Negative |
| SCS | Lys | 0.01475405 | 0.374 | Positive |
| SCS | Arg | 0.93476011 | 0.012 | Positive |
| SCS | Hyp | -0.1519266 | 0.836 | Negative |
| SCS | Total amino acids | 0.5397526 | 0.016 | Positive |

Ala, alanine; Arg, arginine; Asn, asparagine; Asp, aspartic acid; C, catechin; CAF, caffeine; CG, catechin gallate; Cys, cystine; EC, epicatechin; ECG, epicatechin gallate; EGCG, epigallocatechin gallate; EGC, epigallocatechin; GA, gallic acid; GABA, γ-aminobutyric acid; GC, gallocatechin; GCG, gallocatechin gallate; Glu, glutamic acid; Gln, glutamine; Gly, glycine; His, histidine; Hyp, hydroxyproline; Ile, isoleucine; Leu, leucine; Lys, lysine; Met, methionine; Orn, ornithine; Phe, phenylalanine; Ser, serine; TB, theabrownin; TF, theaflavin; TF-3-G, theaflavin-3-gallate; TF-3'-G, theaflavin-3'-gallate; TFs, theaflavins; Thea, theanine; Thr, threonine; TR, thearubigins; Trp, tryptophan; Tyr, tyrosine; Val, valine; β-Ala, β-alanine. AHS represents sourness; PKS and CPS represent composite taste; NMS represents umami; ANS represents sweetness; SCS represents bitterness; $L^*$ represents lightness; $a^*$ represents the degree of greenness (negative)- redness (positive); $b^*$ represents the degree of blueness (negative)-yellowness (positive).

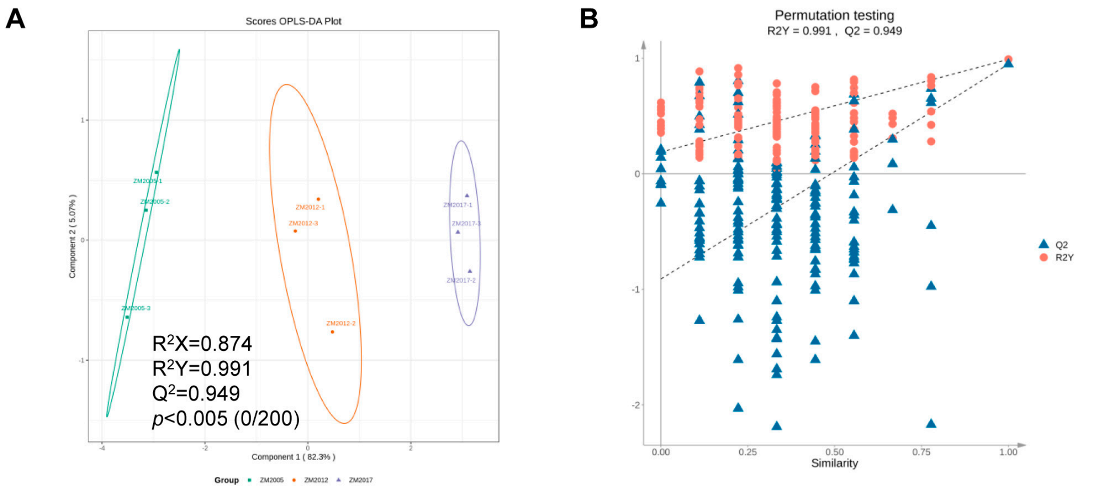


**Figure S1.** Difference analysis of taste and color changes in Liupao tea infusions with different aging durations. (A) Orthogonal partial least squares discriminant analysis (OPLS-DA) score plot. (B) Permutation test of the OPLS-DA model.

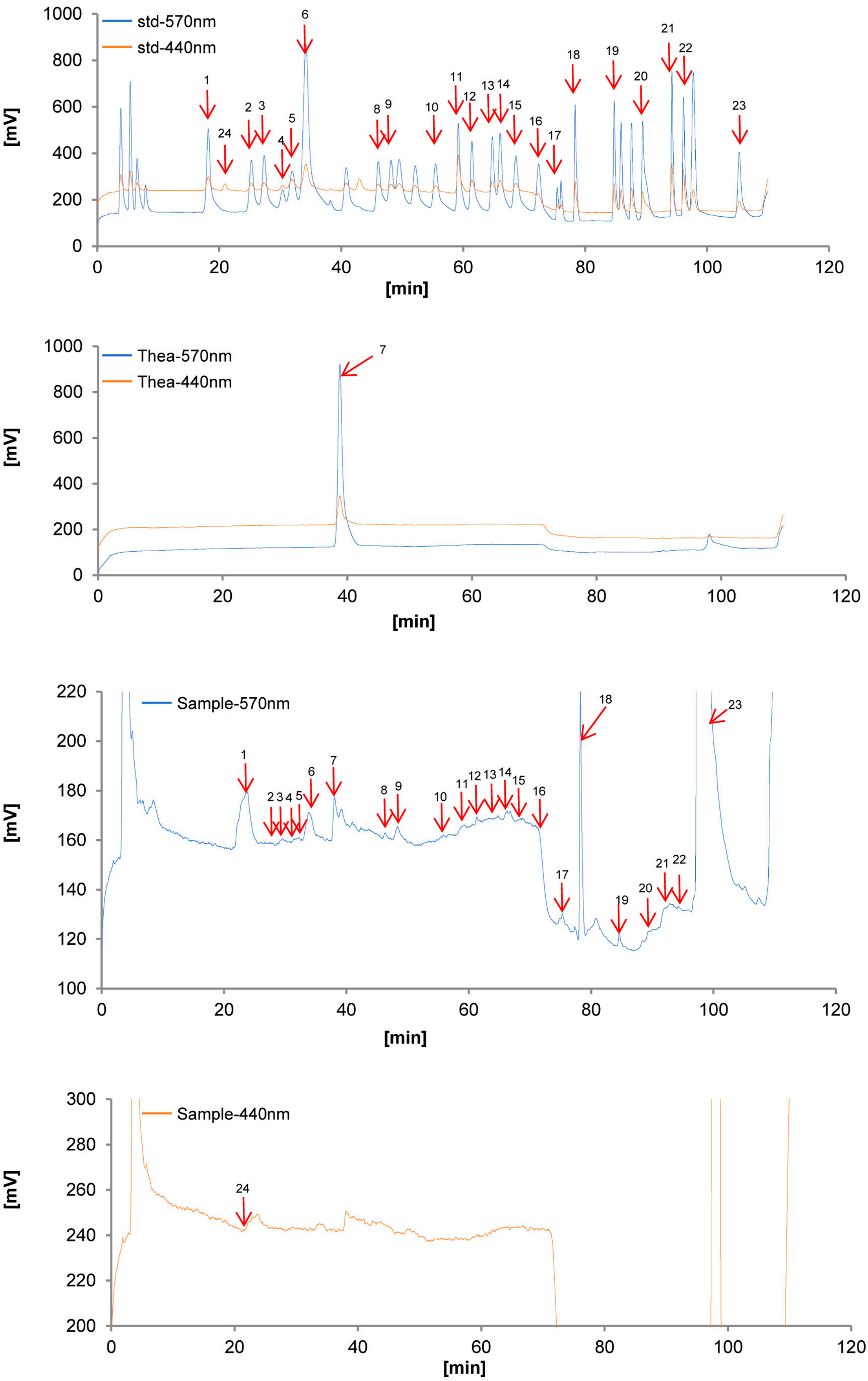


**Figure S2.** Chromatograms of standards and typical tea sample. The numbers represent the following respectively. 1, Aspartic acid (Asp); 2, Threonine (Thr); 3, Serine (Ser); 4, Asparagine (Asn); 5, Glutamic acid (Glu); 6, Glutamine (Gln);7, Theanine (Thea); 8, Glycine (Gly); 9, Alanine (Ala); 10, Valine (Val);

11, Cystine (Cys); 12, Methionine (Met); 13, Isoleucine (Ile); 14, Leucine (Leu); 15, Tyrosine (Tyr); 16, Phenylalanine (Phe); 17, β-Alanine (β-Ala); 18, γ-Aminobutyric acid (GABA); 19, Histidine (His); 20, Tryptophan (Trp); 21, Ornithine (Orn); 22, Lysine (Lys); 23, Arginine (Arg); 24, Hydroxyproline (Hyp).

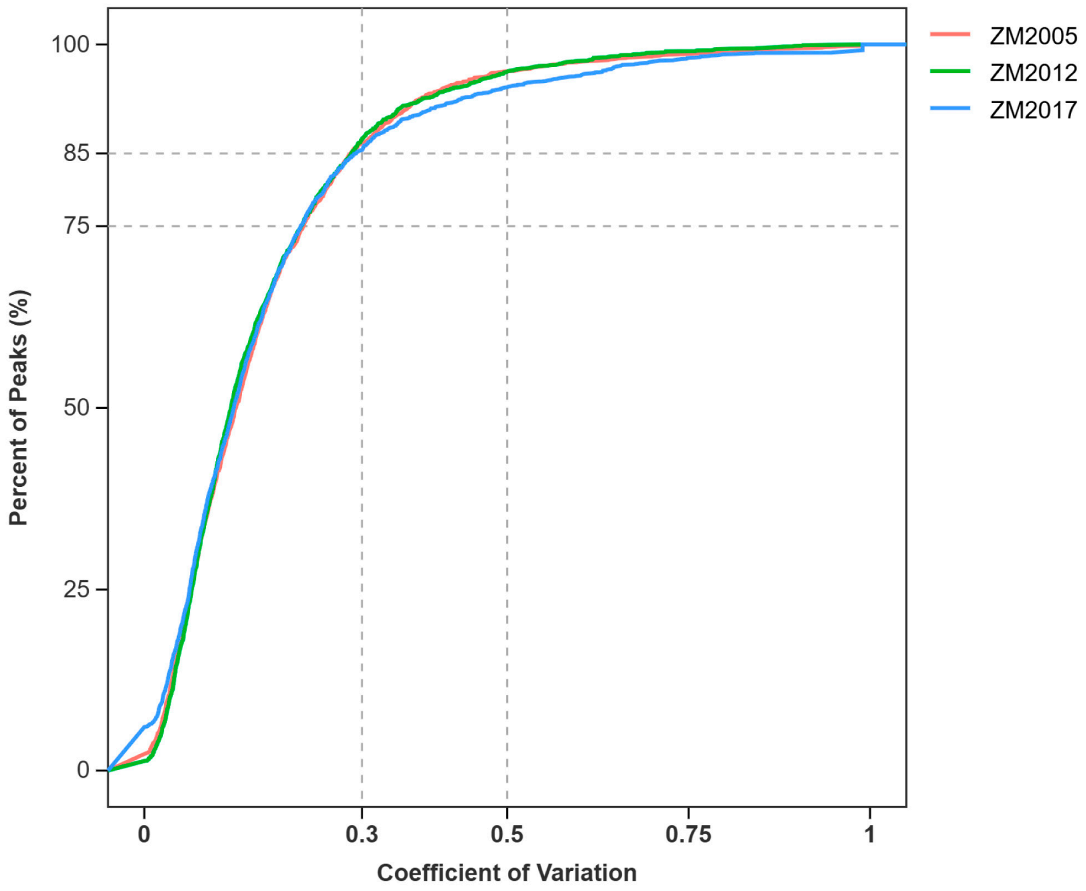


**Figure S3.** Coefficient of variation analysis of metabolites.
